# Amendment of Articles 8, 9, 10, 21 and 78 of the *International Code of Zoological Nomenclature* to expand and refine methods of publication

**DOI:** 10.3897/zookeys.219.3994

**Published:** 2012-09-04

**Authors:** International Commission on Zoological Nomenclature

**Affiliations:** 1 c/o Natural History Museum, Cromwell Road, London SW7 5BD, U.K. (email: iczn@nhm.ac.uk)

**Keywords:** Amendment, archiving, electronic publication, International Code of Zoological Nomenclature, Official Register of Zoological Nomenclature, ZooBank

## Abstract

The International Commission on Zoological Nomenclature has voted in favour of a revised version of the amendment to the *International Code of Zoological Nomenclature* that was proposed in 2008. The purpose of the amendment is to expand and refine the methods of publication allowed by the Code, particularly in relation to electronic publication. The amendment establishes an *Official Register of Zoological Nomenclature* (with ZooBank as its online version), allows electronic publication after 2011 under certain conditions, and disallows publication on optical discs after 2012. The requirements for electronic publications are that the work be registered in ZooBank before it is published, that the work itself state the date of publication and contain evidence that registration has occurred, and that the ZooBank registration state both the name of an electronic archive intended to preserve the work and the ISSN or ISBN associated with the work. Registration of new scientific names and nomenclatural acts is not required. The Commission has confirmed that ZooBank is ready to handle the requirements of the amendment.

In 2008, the International Commission on Zoological Nomenclature (ICZN) published a proposed amendment to the *International Code of Zoological Nomenclature*, 4th edition (ICZN 1999), the primary aim of which was to define a mechanism by which electronic publication of new scientific names and nomenclatural acts could be permitted under the Code (ICZN 2008—BZN **65**: 265-275). The key principles approved by the Commission for drafting the document were: 1) Electronic-only publications should be allowed, if mechanisms can be found that give reasonable assurance of the long-term accessibility of the information they contain; 2) Some method of registration should be part of the mechanism of allowing electronic publication of names and nomenclatural acts; 3) Physical works that are not paper-based (e.g. CD-ROMs, DVDs) should be disallowed (ICZN 2008—BZN **65**: 266). The core principles of the amendment were approved by the International Union of Biological Sciences (IUBS, the governing body for ICZN) in their 2009 general meeting in Cape Town in agreement with Article 78.3 of the Code. Thereafter the details were extensively debated within the Commission, in online forums (especially Taxacom and the ICZN listservers), in the pages of the *Bulletin* (BZN volumes **66-67**, all contributed comments available here: http://iczn.org/content/availability-electronic-publication ), at numerous taxonomic meetings and at an open meeting held in London on 29 October 2011 (summary published in ICZN 2011—BZN **68**: 246-247).

A number of incremental votes were held within the ICZN Council and the Commission to develop consensus wording that satisfied many of the concerns raised during the discussion period. The main decisions reached in these votes were as follows:

1) The changes concerning electronic publication should be effective from the beginning of 2012.

2) The requirement for registration in ZooBank of new scientific names in electronic works was changed to a requirement for registration of the work itself.

3) The requirement that an electronic work be archived was changed to a requirement of intent to archive, with this requirement being satisfied by statement of the intended archive in ZooBank. 

4) A requirement that an ISSN or ISBN (International Standard Serial Number or International Standard Book Number) be included in the ZooBank registration was added.

5) The period during which optical discs such as CD-ROM were acceptable media was changed from “after 2000 and before 2010” to “after 1985 and before 2013”.

In a three-month vote from 9 February to 9 May 2012 the Commissioners voted in favour of the revised amendment, pending a separate vote on the readiness of ZooBank. In a one-month vote from 1 August to 1 September 2012, Commissioners certified that ZooBank was fit for the purpose of handling the requirements of the amendment, thus clearing the last obstacle to allowing electronic publication under the *International Code of Zoological Nomenclature*. The ZooBank development team has established a robust architecture and work flow for registration. During extensive beta testing of ZooBank 3.0 over the last several months, they have demonstrated the ability to respond to problems reported and suggestions made by users. The Commission anticipates that ZooBank will continue to evolve in response to input from the broader community and encourages suggestions for its ongoing development. The text of the revised amendment is published here, with bracketed comments describing the changes from the fourth edition of the Code. 

## Amendment

[Under Article 8 (what constitutes published work), Article 8.1 is modified to accommodate electronic publishing and an example is added. Former Article 8.4 is reformulated as the new 9.2, former Articles 8.5 and 8.6 are simplified and merged under the new Article 8.4, and new Articles 8.5 and 8.6 are introduced. The associated recommendations are revised.]

8.1. **Criteria to be met.** A work must satisfy the following criteria:

8.1.1. it must be issued for the purpose of providing a public and permanent scientific record,

8.1.2. it must be obtainable, when first issued, free of charge or by purchase, and

8.1.3. it must have been produced in an edition containing simultaneously obtainable copies by a method that assures

8.1.3.1. numerous identical and durable copies (see Article 8.4), or

8.1.3.2. widely accessible electronic copies with fixed content and layout.

**Example:** PDF/A (Portable Document Format Archive), described by ISO Standard 19005-1:2005, is a file format that allows content and layout to be preserved unchanged.

[Articles 8.2 and 8.3 are unchanged.]

8.4. **Works issued as physical copies.** Printing on paper and optical disc are the only recognized formats for works issued as physical copies. In addition to fulfilling the requirements of Article 8.1 while not being excluded by Article 9,works issued as physical copies are subject to the following criteria:

8.4.1. Works printed on paper. Before 1986 and after 2012,the only acceptable means of producing physical copies is by printing on paper using ink or toner**.**

8.4.2. Works on optical disc. To be considered published, a work on optical disc must be issued, in read-only memory form, after 1985 and before 2013, and

8.4.2.1. if issued before 2000, must contain a statement that any new name or nomenclatural act within it is intended for public and permanent scientific record and that the work is produced in an edition containing simultaneously obtainable copies, or

8.4.2.2. if issued after 1999, must contain a statement naming at least five major publicly accessible libraries in which copies of the optical disc were to have been deposited.

8.5. **Works issued and distributed electronically.** To be considered published, a work issued and distributed electronically must

8.5.1. have been issued after 2011,

8.5.2. state the date of publication in the work itself, and

8.5.3. be registered in the *Official Register of Zoological Nomenclature* (ZooBank) (see Article 78.2.4) and contain evidence in the work itself that such registration has occurred.

**Examples.** Evidence of registration is given by stating information that would be known only if the registration has occurred, such as the exact date of registration or the registration number assigned to the work or to a new name or nomenclatural act introduced in the work. A work issued as a PDF may contain the registration number as an embedded hyperlink. Even if the registration number is not visible in the normal viewing mode of the file or when the work is printed from the file, it is deemed to be cited in the work itself because the text of the hyperlink can easily be revealed using standard software for viewing PDFs.

8.5.3.1. The entry in the *Official Register of Zoological Nomenclature* must give the name and Internet address of an organization other than the publisher that is intended to permanently archive the work in a manner that preserves the content and layout, and is capable of doing so. This information is not required to appear in the work itself.

8.5.3.2. The entry in the *Official Register of Zoological Nomenclature* must give an ISBN for the work or an ISSN for the journal containing the work. The number is not required to appear in the work itself.

8.5.3.3. An error in stating the evidence of registration does not make a work unavailable, provided that the work can be unambiguously associated with a record created in the *Official Register of Zoological Nomenclature* before the work was published.

**Examples.** The following are examples of admissible errors: In preparing a manuscript an author accidentally deletes the final digit of the registration number. An author states the wrong date of registration forgetting that ZooBank uses Coordinated Universal Time rather than local time. An author registers two works that are in review for publication and accidentally uses the same ZooBank number in both published versions.

The following are examples of inadmissible errors: An author, in preparing a manuscript for publication, states that day’s date for the registration date, intending to register it later that day but forgetting to do so. The author discovers the omission after the work is published and immediately registers it; because registration occurred after publication, the work is not available. A publisher discovers errors in a work and reissues it to correct those errors, but instead of registering the new edition, uses the original ZooBank number; the revised edition is not available because it was not separately registered.

8.6. **New methods of publication and archiving.** The Commission may issue Declarations to clarify whether new or unconventional methods of production, distribution, formatting or archiving can produce works that are published in the meaning of the Code.

[Article 8.7 is unchanged. Recommendation 8A is modified, and new Recommendations 8B, 8C, 8D and 8H are added. The former 8B is deleted, the former 8C is modified and renumbered as 8E and the former 8D and 8E becomes the new 8F and 8G but are otherwise unchanged.]

**Recommendation 8A. Wide dissemination.** Authors have a responsibility to ensure that new scientific names, nomenclatural acts, and information likely to affect nomenclature are made widely known. Authors can accomplish this by publishing in appropriate scientific journals or well-known monographic series, by entering new names and nomenclatural acts into the *Official Register of Zoological Nomenclature* (ZooBank), and by sending copies of their works to the *Zoological Record*.

**Recommendation 8B. Minimum edition of printed works.** A work on paper should be issued in a minimum edition of 25 copies, printed before any is distributed.

**Recommendation 8C. Electronic works.** Electronic works should be structured to allow automated indexing and data extraction and should include actionable links to external resources (such as embedded hyperlinks to records in the *Official Register of Zoological Nomenclature*), where appropriate.

**Recommendation 8D.** **Content immutable.** The content of a work is immutable once it is published. Corrections should be made through notices of errata or other separate publications. Second or other additional printings of a work should be clearly labeled as such, with date of publication stated in the work, even if no changes have been introduced.

**Recommendation 8E. Public accessibility of published works.** Copies of published works that contain new scientific names or nomenclatural acts, or information likely to affect nomenclature, should be permanently conserved in or by libraries that make their holdings publicly accessible.

**Recommendation 8H. Archiving encouraged.** Authors are encouraged to ensure that their electronic works are archived with more than one archiving organization. Archiving organizations utilized for registered works should have permanent or irrevocable license to make a work accessible should the publisher no longer do so.

[Under Article 9, new Articles 9.2, 9.3 and 9.9 are added. Former Articles 9.2 through 9.6 are renumbered as 9.4 to 9.8. The former 9.7, 9.8 and 9.8 are reformulated as the new 9.12, 9.11 and 9.10, respectively. An example is added for 9.12 and Recommendation 9A is rephrased.]

**Article 9. What does not constitute published work.** Notwithstanding the provisions of Article 8, none of the following constitutes published work within the meaning of the Code:

9.1. after 1930, handwriting reproduced in facsimile by any process;

9.2. after 1985, works produced by hectographing or mimeographing;

9.3. before 1986 and after 2012, works issued on optical discs;

9.4. photographs as such;

9.5. proof sheets;

9.6. microfilms;

9.7. acoustic records made by any method;

9.8. labels of specimens;

9.9. preliminary versions of works accessible electronically in advance of publication (see Article 21.8.3);

9.10. materials issued primarily to participants at meetings (e.g. symposia, colloquia, congresses, or workshops), including abstracts and texts of presentations or posters;

9.11. text or illustrations distributed by means of electronic signals (e.g. via the Internet), except those fulfilling the requirements of Articles 8.1 and 8.5.

9.12. facsimiles or reproductions obtained on demand of an unpublished work [Art. 8], even if previously deposited in a library or other archive.

**Example:** A Ph.D. thesis that was distributed only to members of the student’s thesis committee is listed for sale in the catalogue of a print-on-demand publisher. The print-on-demand work is a reproduction of the thesis. Because the thesis was an unpublished work in its original form, it remains unpublished. If an editorial process was evident in converting the work to print-on-demand form (e.g., change to single spacing, repagination, addition of running headers), it might be considered published.

**Recommendation 9A. Avoidance of new names and acts in meeting abstracts.** Authors should not include new names and nomenclatural acts in abstracts of papers or posters to be presented at meetings. This avoids the appearance that they are published and prevents inadvertent publication if the abstracts are widely distributed. (For disclaimer of abstracts volumes, see Recommendation 8G.)

[Changes to Article 10 (Criteria of Availability) proposed in the original draft of the Amendment have been removed, except that Recommendation 10B is modified and placed after Article 10.7.]

**Recommendation 10B. Registration of names encouraged.** Authors are encouraged to include registration numbers from the *Official Register of Zoological Nomenclature* for new names and nomenclatural acts introduced in their publications, and to register names and acts that have been previously published.

[Under Article 21 (determination of date), Articles 21.7 and 21.8 are modified and Article 21.9 is added.]

21.7. **Date not specified.** If the date of publication is not specified in a work the earliest day on which the work, or a part of it, is demonstrated to be in existence as a published work is to be adopted as the date of publication of the work or of that part.

21.7.1. In the absence of evidence as to day, the provisions of Article 21.3 apply.

21.7.2. Works issued as electronic copies are required to state a date of publication (Article 8.5.2), even if incompletely specified (Article 21.3).

21.8. **Advance distribution of separates and preprints.** Advance distribution of separates or preprints affects date of publication as specified by the following criteria:

21.8.1. Before 2000, an author who distributed separates in advance of the specified date of publication of the work in which the material was published thereby advanced the date of publication.

21.8.2. The advance issue of separates after 1999 does not advance the date of publication, whereas preprints on paper, unambiguously imprinted with their own date of publication, are published works from the date of their issue, if they fulfil the criteria for publication in Article 8 and are not excluded by Article 9 (see Glossary: “separate”, “preprint”).

21.8.3.Some works are accessible online in preliminary versions before the publication date of the final version. Such advance electronic access does not advance the date of publication of a work, as preliminary versions are not published (Article 9.9).

21.9. **Works issued on paper and electronically.** A name or nomenclatural act published in a work issued in both print and electronic editions takes its date of publication from the edition that first fulfilled the criteria of publication of Article 8 and is not excluded by Article 9.

[Under Article 78 (powers and duties of the Commission), Article 78.2.4 is added to allow establishment of the *Official Register*.]

78.2.4. The Commission may establish and maintain an *Official Register of Zoological Nomenclature* (ZooBank), to record essential information about works, names and nomenclatural acts. The *Official Register of Zoological Nomenclature* may be maintained in electronic or paper form. The *Official Lists* and *Official Indexes* may be maintained in the *Official Register*.

[The following terms are added to the Glossary.]

archive, n. A depository for works (q.v.); v. to place a work in an archive with the intent that it be permanently preserved there.

*Official Register*, n. An abbreviated title for the *Official Register of Zoological Nomenclature* [Article 78.2.4], maintained by the Commission to record information about works, names and nomenclatural acts (see ZooBank).

optical disc, n. a laser-readable data storage medium. Compact disc read-only memory (CD-ROM) and digital video disc read-only memory (DVD-ROM) are optical disc formats that could be used to produce available works after 1985 and before 2013 (Article 8.4.2).

publication, electronic, n. A publication issued and distributed by means of electronic signals.

register, v. To enter into the *Official Register* information about a work, name, author, nomenclatural act, or other item tracked for purposes of zoological nomenclature.

registration number, n. A unique identifying number or alpha-numeric code assigned in the *Official Register* to a particular item.

ZooBank, n. The online version of the *Official Register of Zoological Nomenclature*.

## Discussion

The reasoning behind the five main changes to the amendment summarized in the Introduction is detailed here. Further information about ZooBank in its role as the *Official Register of Zoological Nomenclature* will be published in subsequent issues of the *Bulletin of Zoological Nomenclature*.

*Retroactivity*. Although the Amendment is retroactive to 1 January 2012, there are no works that fulfilled the requirements of the amendment as of that date. The live version of ZooBank did not support the fields for statement of intended archive and ISSN or ISBN until later in the year (September 2012), because the requirements had not been established by vote of the Commission. Some Commissioners therefore felt that it was better to have a start date of 1 January 2013. That, however, raised a different problem, that ZooBank would have to support the required fields in advance of that date in order to allow pre-registration. Either way, electronic journal articles might appear that contained ZooBank registration numbers that were nonetheless not published under the Code. The majority of Commissioners deemed it better to allow electronic publication sooner than later.

*Registration of works*. The shift to registration of works instead of registration of names addressed a problem caused by requiring registration of names but not of acts (as originally proposed): a work could have names that were not available because they were not registered, but nomenclatural acts in the same work would be available, which would be confusing. The alternative approach, to also require registration of acts in electronic works, was problematic, as it would be easy for authors to forget to register acts such as first reviser’s choices in situations where the Code does not currently require a statement that an act has occurred. The shift to registering works lets registration of names and acts proceed on a voluntary basis, which gives more time to fully develop those functions in ZooBank, and allows more informed decision-making if such registrations are proposed to be mandatory in the future.

*Intent to archive*. The original proposal asked for archiving within one year, which created a “limbo period” during which it would not be known if the archiving requirement had been fulfilled. The change to requiring “intent to archive”, analogous to intent to deposit a holotype (Article 16.4), eliminates this uncertainty. ZooBank provides a list of accepted archives and stores archive information for journals where such is known. If a user leaves the archive field blank, ZooBank warns that a statement of the intended archive is required for electronic publication. Users can suggest archives to be added to the accepted list in ZooBank.

*ISSN/ISBN*. A majority of Commissioners thought it desirable to add a requirement that an electronic work have an ISSN or ISBN to be registered in ZooBank. Reasons in favour included consistency with the new botanical rule for electronic publications and likelihood that works with ISSN and ISBN would be deposited in national archives, as deposition is required in some countries. Reasons against included that ISSN and ISBN give no assurance of quality while increasing costs and that some outlets for taxonomic monographs have not traditionally used ISBN. The new Article 8.5.3.2 does not require that the ISSN or ISBN appear in the work.

*Status of optical discs*. The Commission changed the date ranges when optical discs could be used as a medium of publication under certain conditions to avoid retroactively disallowing works that formerly had published status. The discussion of the proposed amendment in BZN **65**: 275 noted that the Commission was not aware of works on CD-ROM issued before 2000 in compliance with the requirements of the former Article 8.5. A few such works have now been brought to the Commission’s attention, so the “after 1985” time frame has been restored. Similarly, some works on optical disc have been produced during 2012 in compliance with the former Article 8.6. Rather than have all parts of the amendment come into effective on 1 January 2012, the Commission has allowed publication on CD-ROM to continue through 2012 under the conditions specified in Article 8.4.2.

## The Vote

The Commission voted as follows on the final version of the amendment as reproduced above:

For: 23

Against: 3

Abstain: 1

and as follows on the proposition that ZooBank, as available in August 2012, is fit for the purpose of handling the requirements of the amendment:

For: 23

Against: 4
